# Early‐life patterns of growth are linked to levels of phenotypic trait covariance and postfledging mortality across avian species

**DOI:** 10.1002/ece3.8231

**Published:** 2021-11-05

**Authors:** Loren Merrill, Todd M. Jones, Jeffrey D. Brawn, Michael P. Ward

**Affiliations:** ^1^ Department of Natural Resources and Environmental Sciences University of Illinois at Urbana‐Champaign Urbana Illinois USA; ^2^ Illinois Natural History Survey Prairie Research Institute University of Illinois at Urbana‐Champaign Champaign Illinois USA

**Keywords:** canalization, developmental flexibility, early‐life stress, nest predation, phenotypic correlation, trait covariance

## Abstract

Life history studies have established that trade‐offs between growth and survival are common both within and among species. Identifying the factor(s) that mediate this trade‐off has proven difficult, however, especially at the among‐species level. In this study, we examined a series of potentially interrelated traits in a community of temperate‐zone passerine birds to help understand the putative causes and consequences of variation in early‐life growth among species. First, we examined whether nest predation risk (a proven driver of interspecific variation in growth and development rates) was correlated with species‐level patterns of incubation duration and nestling period length. We then assessed whether proxies for growth rate covaried with mean trait covariance strength (i.e., phenotypic correlations (*^r^p)*, which can be a marker of early‐life stress) among body mass, tarsus length, and wing length at fledging. Finally, we examined whether trait covariance strength at fledging was related to postfledging survival. We found that higher nest predation risk was correlated with faster skeletal growth and that our proxies for growth corresponded with increased trait covariance strength (*^r^p)*, which subsequently, correlated with higher mortality in the next life stage (postfledging period). These results provide an indication that extrinsic pressures (nest predation) impact rates of growth, and that there are costs of rapid growth across species, expressed as higher mean *^r^p* and elevated postfledging mortality. The link between higher levels of trait covariance at fledging and increased mortality is unclear, but increased trait covariance strength may reflect reduced phenotypic flexibility (i.e., phenotypic canalization), which may limit an organism's capacity for coping with environmental or ecological variability.

## Graphical abstract

Our manuscript proposes that constrained developmental flexibility is a mechanism mediating the tradeoff between growth and survival at the among‐species level. This tradeoff has received considerable attention from developmental biologists, ecological physiologists, and evolutionary biologists among others, but evidence for universal mechanisms has been lacking. Our findings present evidence that species with more rapid growth rates exhibit higher levels of trait covariance (an indication of increased trait canalization and reduced developmental flexibility), and that higher levels of trait covariance result in elevated rates of mortality.

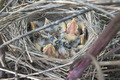

## 1 INTRODUCTION

**FIGURE 1:**
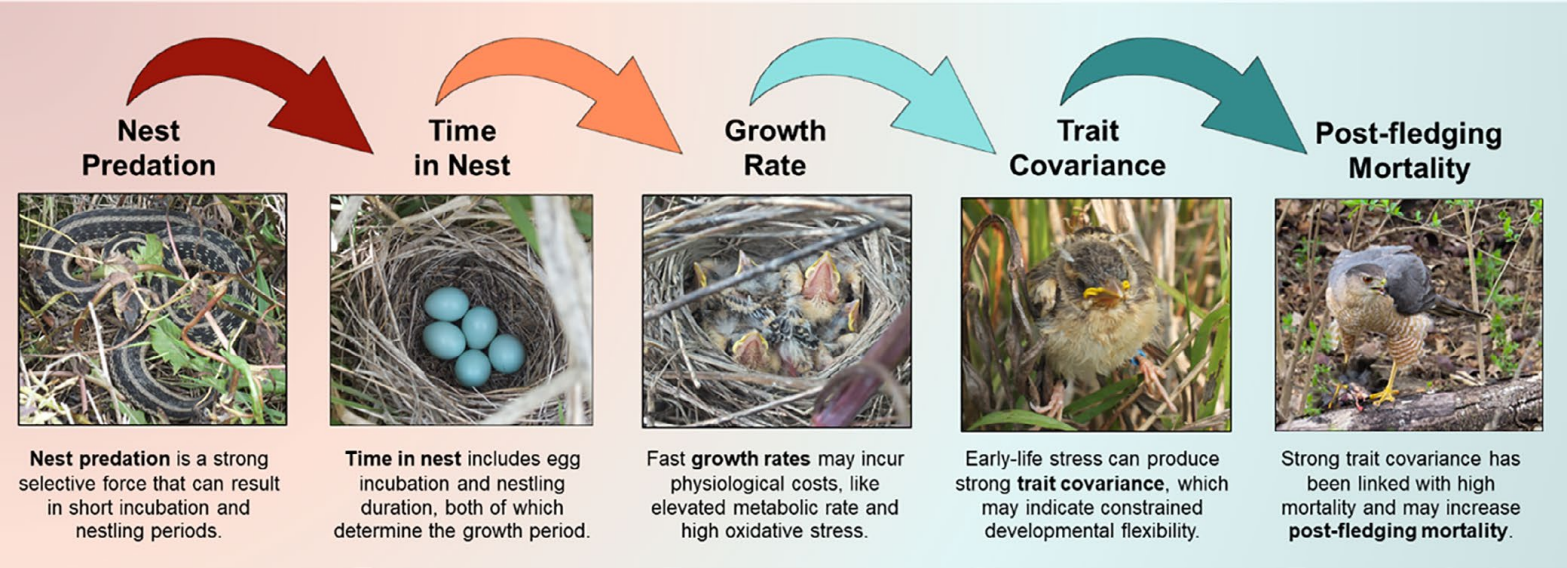
Theoretical framework for how nest predation pressure can impact growth and development, which impacts developmental flexibility, and the next life stage mortality rates. Nest predation pressure is a strong selective force that can result in shorter incubation and nestling periods and faster offspring growth (Bosque & Bosque, 1995; Martin, 1995; Martin et al., 2018; Remeš, 2007; Remeš & Martin, 2002; Remeš et al., 2020; Ton & Martin, 2020). Time in nest includes egg incubation and nestling duration, both of which determine the growth period. Fast growth may be necessary for species under high nest predation risk, but it may entail physiological and physical costs (Arendt, 1997; DeBlock & Stoks, 2008; Janssens & Stoks, 2018; Tarry‐Adkins et al., 2009; Xie et al., 2015). If rapid growth is costly for nestlings, it may result in elevated levels of trait covariance strength, indicative of constrained developmental flexibility (Merrill & Grindstaff, 2018; Van Dongen, 2006). Stronger trait covariance levels have been associated with increased early‐life mortality (Merrill & Grindstaff, 2018) and may influence postfledgling mortality rates

Most endothermic vertebrates exhibit determinate growth and relatively fixed, species‐specific growth rates. These growth rates are shaped by the trade‐offs between extrinsic pressures and a suite of intrinsic costs and constraints associated with fast growth (Arendt, 1997) (Figure 1). Rapid growth may also be beneficial because it allows an organism to gain a competitive advantage for access to food resources, attain reproductive size at an earlier age, and escape stage‐dependent predation, among others (reviewed in Arendt, 1997). However, we know from studies conducted at the intraspecific level that there are numerous physiological costs associated with elevated rates of growth (DeBlock & Stoks, 2008; Janssens & Stoks, 2018; Tarry‐Adkins et al., 2009; Xie et al., 2015). Moreover, species with more rapid growth rates also have higher metabolic rates and reduced longevity (Rollo, 2002; Ricklefs, 2006, but see Martin et al., 2015), suggesting that there may be intrinsic constraints on growth. Despite these broad patterns within and among species, it has proven challenging to identify the factors limiting growth rates at the interspecific level due to each species’ presumed capacity to evolve mechanisms for coping with the physiological costs of rapid growth (sensu Metcalfe & Monaghan, 2003). Oxidative damage has been proposed as the mechanism of this growth‐longevity trade‐off (Dowling & Simmons, 2009; Monaghan et al., 2009), but the evidence in support of this relationship at the interspecific level remains inconclusive (reviewed in Selman et al., 2012).

If rapid growth is itself a stressor, there may be identifiable markers of this stress beyond elevated metabolic activity and reduced longevity in species that exhibit more rapid rates of growth and development in early life. One such potential marker is the strength of associations among traits and within traits over time (i.e., phenotypic trait covariance – *^r^p*). Phenotypic trait covariance indicates how correlated two traits are among individuals. For instance, if values for two traits are plotted against one another (e.g., wing length on the *x*‐axis and body mass on the *y*‐axis), points may be expected to tightly covary (a high correlation, or high *^r^p*) or may only be loosely correlated (a low correlation, or low *^r^p*). Phenotypic trait covariance strength has proven to be a powerful tool for uncovering costs associated with developmental stress (Careau et al., 2014; Hebert et al., 1994; Killen et al., 2013; Merrill & Grindstaff, 2018; Merrill et al., 2017). Recent work in zebra finches (*Taeniopygia guttata*), for example, documented that stress during development resulted in near‐universal increases in trait covariance strength for a broad range of physiological and morphological traits (e.g., mass, tarsus, wing length, and concentrations of corticosterone, antibodies, and haptoglobin; Merrill & Grindstaff, 2018). Moreover, higher levels of trait covariance had measurable costs, as finches with greater trait covariance died earlier (Merrill & Grindstaff, 2018). Stronger trait associations may reflect more constrained developmental trajectories (i.e., phenotypic canalization; Merrill & Grindstaff, 2018; Van Dongen, 2006) and thus a reduced capacity for developmental flexibility (*sensu* Gianoli & Palacio‐Lopez, 2009). There is a rich body of research examining long‐term (e.g., macro‐evolutionary) and developmental (e.g., plasticity) processes that impact levels of trait covariance among functionally related traits, and much of this work falls within the realm of phenotypic integration (Armbruster et al., 1999; Pigliucci, 2003; Schlichting, 1989). It is not yet clear, however, how well this concept explains emerging patterns of trait covariance in work examining the effects of different early‐life conditions. As documented previously (Careau et al., 2014; Hebert et al., 1994; Killen et al., 2013; Merrill & Grindstaff, 2018; Merrill et al., 2017), challenging early‐life conditions can result in more positive trait covariance. However, they can also result in more negative trait covariance, such that two traits are inversely correlated with one another (Merrill & Grindstaff, 2018; Merrill et al., 2017). We do not yet know what mechanisms drive the stronger levels of trait covariance, although glucocorticoids and reactive oxygen species are two plausible factors that may link early‐life challenges to altered phenotypic expression (Dowling & Simmons, 2009; Merrill & Grindstaff, 2018; Monaghan et al., 2009). Moreover, it remains unclear whether the patterns of trait covariance documented within species would also occur at the among‐species level.

To better understand the factors shaping interspecific growth and development rates and the potential costs associated with rapid growth (Figure 1), we explored associations among early‐life mortality, incubation and nestling durations (proxies for growth rates), and morphological trait covariance strength in a community of passerine bird species that experiences broadly different rates of nest predation, and exhibits a wide range of developmental periods (Table 1). Specifically, we investigated the following questions:

1. Do species‐level nest mortality rates covary with (A) egg incubation duration and nestling duration, and (B) interspecific patterns of growth?
2. Are interspecific patterns of growth associated with prefledging trait covariance strength (*^r^p*) among morphological traits?
3. Does prefledging *^r^p* strength predict postfledging mortality among species?

**TABLE 1:** Summary statistics for nest mortality, nestling trait covariance, cumulative postfledging mortality, and length of the nestling period for 21 songbird species breeding in grasslands/shrublands of East‐central Illinois, USA, 2014–2019

Species	Alpha Code	Scientific Name	Nest Type	No. Nests	No. Nestlings Sampled	No. Nestlings Postfledge^a^	Nest Daily Mortality Rate	Cumulative Postfledging Mortality^b^	Nestling period (days)^c^
American Robin	AMRO	*Turdus migratorius*	Cup	19	8	—	0.078	—	14.1
Brow‐headed Cowbird	BHCO	*Molothrus ater*	Cup	—	53	39	—	0.740	9.9
Blue Grosbeak	BLGR	*Passerina caerulea*	Cup	6	4	—	0.072	—	11.0
Brown Thrasher	BRTH	*Toxostoma rufum*	Cup	130	98	28	0.059	0.542	11.7
Blue‐winged Warbler	BWWA	*Vermivora cyanoptera*	Cup	7	9	—	0.113	—	10.0
Carolina Chickadee	CACH	*Poecile carolinensis*	Cavity	8	19	—	0.020	—	17.6
Chipping Sparrow	CHSP	*Spizella passerina*	Cup	20	15	—	0.088	—	10.4
Common Yellowthroat	COYE	*Geothlypis trichas*	Cup	165	136	37	0.080	0.408	9.2
Dickcissel	DICK	*Spiza americana*	Cup	454	298	102	0.080	0.667	8.2
Eastern Bluebird	EABL	*Sialia sialis*	Cavity	168	348	32	0.015	0.246	17.3
Eastern Phoebe	EAPH	*Sayornis phoebe*	Cup^d^	37	67	8	0.014	0.239	16.4
Eastern Towhee	EATO	*Pipilo erythrophthalmus*	Cup	53	17	—	0.089	—	9.8
Field Sparrow	FISP	*Spizella pusilla*	Cup	322	157	28	0.087	0.467	8.7
Gray Catbird	GRCA	*Dumetella carolinensis*	Cup	136	95	34	0.065	0.543	10.8
House Wren	HOWR	*Troglodytes aedon*	Cavity	73	195	—	0.010	—	16.0
Indigo Bunting	INBU	*Passerina cyanea*	Cup	139	92	28	0.064	0.283	10.1
Northern Cardinal	NOCA	*Cardinalis cardinalis*	Cup	100	45	—	0.076	—	10.2
Red‐winged Blackbird	RWBL	*Agelaius phoeniceus*	Cup	271	104	41	0.081	0.488	11.0
Tree Swallow	TRES	*Tachycineta bicolor*	Cavity	78	199	—	0.009	—	18.4
Yellow‐breasted Chat	YBCH	*Icteria virens*	Cup	34	16	—	0.066	—	9.1
Yellow Warbler	YEWA	*Setophaga petechia*	Cup	8	7	—	0.088	—	10.0

^a^ Number of nestlings tagged and tracked during the postfledging period and from which cumulative postfledging estimates are derived.

^b^ Cumulative postfledging mortality was derived from daily survival estimates based on fledgling age, multiplied out to 28 days postfledging.

^c^ Average length of the nestling period determined by the age at which each juvenile fledged the nest.

^d^ Eastern phoebes use an open‐cup nest, but it is constructed of mud and placed under an overhanging structure such that it is heavily protected from predators. Nest survival and nestling period align more closely with cavity nesters than open‐cup nesters.

As outlined in Figure 1, we predicted that species with higher rates of nest predation would be under increased selective pressure to leave the nest at an earlier age (fledge early), and thus exhibit more rapid rates of growth and development (Bosque & Bosque, 1995; Martin, 1995; Remeš & Martin, 2002). We also predicted that if more rapid rates of growth are physiologically stressful, *^r^p* strength would positively covary with interspecific rates of growth. Finally, we predicted that species with higher *^r^p* would experience higher rates of postfledging mortality.

## 2 MATERIALS AND METHODS

### 2.1 Study sites and species

We studied 21 species of grassland/shrubland nesting songbirds (see Table 1 for species list and sample sizes) in East‐central Illinois, USA (~40°N), between 2014 and 2019. All species examined are in the order Passeriformes and therefore have altricial young, thereby controlling for differences in water content, and subsequent variation in growth patterns between altricial and precocial offspring (Ricklefs, 2003). Nest predation is a major source of nest failure for many species with altricial offspring, but this can vary by nest‐type. Our species fall into two general categories of nesters: open cup and cavity. Cavity nesters generally exhibit protracted nestling periods, which is often attributed to reduced nest predation pressure (Martin & Li, 1992). We included cavity nesters and open‐cup nesters to ensure that we had a broad range of nest predation risks (0.009–0.133 daily mortality) and nestling development periods (8.2–18.4 days; Table 1).

### 2.2 Nest mortality, nestling growth, and development

We located songbird nests from April through August by systematically searching appropriate habitat and observing behavioral cues of adults (e.g., adults returning to the nest to incubate of feed offspring, nest building). To document nest life‐history traits such as incubation and nestling period length, and determine nest fate (fledge/fail), we checked nests every 3 to 6 days (average 3) during the incubation period and much of the nestling period, and every 1 to 2 days as the predicted date of fledging approached. We assumed nest failure/predation when all contents of the nest (eggs/nestlings) disappeared before the predicted day of fledging and we did not observe adults feeding fledglings.

We measured nestlings on the day of fledging, at which point we weighed them (± 0.01 g), recorded wing length (± 0.5 mm) and tarsus length (± 0.01 mm), and banded them with a U.S. Geological Survey metal band. All juveniles in this study were banded and sampled by the same researcher. We assessed trait covariance at fledging rather than an arbitrary day posthatch (e.g., Day 7), as a way to standardize sampling across all the study species. For example, a Field Sparrow (*Spizella pusilla*) is at a different developmental stage on Day 7 compared to an Eastern Bluebird (*Sialia sialis*). We therefore determined that assigning a life stage (fledging) as the reference point was most useful. Indeed, the factors that affect growth and development of juveniles up to the point of fledging can have important carryover effects on subsequent survival during the postfledging period (i.e., “pre‐ to postfledging carryover effects”; Jones & Ward, 2020; Martin et al., 2018). Thus, by sampling traits at fledging we can assess impacts of early‐life conditions on nestling growth and development, while also linking those changes to subsequent juvenile survival/fitness (interspecific postfledging mortality (PFM) rates). We did our best to limit the potential effect of force fledging while capturing nestlings. In cases where a nestling force fledged, we were able to recapture the nestling and return it to the nest so it could leave on its own. We placed a bag over all nestlings once we returned them to the nest, for 5 to 10 min, which was usually long enough to calm them down and for them to remain in the nest. Consequently, we had a number of occasions where nestlings were force fledged, captured, processed, and then returned to the nest and were re‐sampled (in the nest) the next day (sensu Jones & Ward, 2020). In this way, we were able to capture and sample nestlings as close to fledging as possible, which was our point of interest.

We examined two measures of “growth time” to explore whether the period of nestling growth or the combination of embryonic and nestling growth periods better predicted trait covariance strength. To correct for variation among species in body size at fledging, we also estimated size‐adjusted indices of growth for each bird in which size at fledging was divided by the number of days from hatch to fledge (posthatch growth), or the number of days from incubation initiation to fledge (postlay growth). The duration of egg incubation may better standardize the developmental starting point from the fertilized gamete. It is important to note that we use these temporal variables, and size‐adjusted measures for growth because birds were only measured once. As such, our measures serve as proxies for traditional estimates derived from growth curves with multiple measures per individual. However, we believe that the duration of both incubation period and the nestling period should provide meaningful information on rates of growth and development during those respective periods. Incubation duration should provide a proxy for embryonic growth rate (Ricklefs, 2010), and there is evidence that slower rates of embryonic growth are positively linked to increased nestling immune function and reduced adult mortality (reviewed in Ricklefs et al., 2017). Martin (2015) showed a strong relationship between nestling period and growth rate across numerous temperate and tropical species, indicating that our nestling period data should provide a reasonable estimate for nestling growth rates.

Due to incomplete data on incubation duration for the nests we studied, we used published accounts from the literature to estimate each species’ incubation duration (Rodewald, 2015). When possible, we used values from studies conducted at similar latitudes to where we worked. When data from similar latitudes were not available, we used a mean value of published incubation ranges. We quantified fledging age as the number of days between when the brood hatched and when each nestling left the nest (we occasionally observed nestlings in the same brood leave the nest on different days).

### 2.3 Monitoring fledgling mortality

For nine of our 21 focal species (Table 1), we quantified PFM over the first 28 days out of the nest. We randomly selected one nestling per brood (except for dickcissels (*Spiza americana)* where 1 to 3 individuals were tagged per brood as part of another study; Jones et al., 2017) to which we fitted a small (0.3 to 1.0 g, depending on a species’ size) radio‐transmitter via a leg harness constructed with elastic bead cord—which allows for the harness to expand as juveniles grow. We attempted to locate radio‐tagged juveniles every 1 to 3 days after fledging until they either dispersed, died, or their radio's battery failed. We located tagged juveniles by homing into their signal with a handheld Yagi and receiver, and if we were unable to detect a signal, we spent at least 30 min in adjacent habitat (~400 m) in an attempt to re‐locate individuals.

### 2.4 Statistical analyses

We quantified nest daily mortality rates (DMR(s)) for each species (except for brown‐headed cowbirds, in which offspring are placed in different host nests and therefore experience differences in nest survival) using the logistic exposure method (Shaffer, 2004) in SAS. Based on extensive nest camera work conducted on the shrubland bird community at our field site and at similar, nearby field sites, we attributed most (>95%) of nest failures in our study to predation (primarily snakes, raccoons, squirrels, and weasels; Chiavacci et al., 2018; Merrill et al., 2019). Therefore, DMR should represent an accurate level of nest predation risk for each species.

We used multi‐state models in program MARK (White and Burnham 1999) to estimate cumulative rates of PFM (i.e., the probability of a fledgling dying during the postfledging period) for the nine focal species that received radio‐transmitters. Following methods in Jones et al. (2017), we first assigned each fledgling observation to either an alive or dead state. For all models, we then fixed the survival probability to one, transitions to absorbing states (e.g., dead to alive, dead to dead) to zero, and estimated fledgling mortality rates using transition probabilities (Ψ) from the alive to the dead state. Past research on the postfledging period has identified age as the main predictor of fledgling survival in birds (Cox et al., 2014). Thus, before we derived our cumulative mortality rates from daily survival rates DSR(s), we refined our model by determining how the probability of fledgling mortality was best described by age. For each species, we examined 10 models with a priori hypotheses of age structure predicting DSRs of fledglings. For each species, we used seven “standard” hypotheses (same among species) based on age structures of past postfledging studies, a null (constant rate) model, and two models which we based on the observed timing of fledgling death (models which differ among species; see Jones & Ward, 2020 for more details of age structures). We used Akaike's information criteria adjusting for small samples size (AICc) for model selection (Burnham & Anderson, 2002), then used age specific DSRs from our top model to derive a cumulative mortality rate estimate (one minus the cumulative survival rate) for each species. We estimated cumulative rates up to 28 days postfledging, a point past which the vast majority (>98%) of our fledglings survive, and thus reflects an appropriate, accurate, and comparable point to estimate PFM among species (Jones & Ward, 2020).

We examined associations between three morphological traits: mass, wing length, and tarsus length. For one species (dickcissels), wing length was not recorded from 2014 to 2015, limiting associations in those years to between mass and tarsus length. To assess the phenotypic correlations, we calculated the phenotypic correlation coefficient (*^r^p*) for each trait‐by‐trait comparison. For each species, we then calculated the average *^r^p* across the three associations as an overall measure of trait covariance strength. Of the nestlings measured (Table 1), we removed several outlies because they were either significantly smaller than any other nestlings of the same species or because they were extreme outliers based on both trait x trait associations (e.g., if mass was removed, it was because that bird's mass was an outlier for both mass x tarsus length *and* mass × wing length associations). Including these values in the analyses did not impact results qualitatively, but these individuals or individual traits had a disproportionate effect on correlation coefficient values relative to all other individuals of the same species. Importantly, we note that our correlations are not confounded by potential differences in the stage of growth (i.e., still growing vs. fully grown) for each trait, as in all species juveniles had fully grown tarsi but had yet to reach adult levels of mass and wing length.

We tested if nest predation pressure (as defined by DMR) was linked to interspecific rates of growth. We did this by first examining the associations between nest DMR and the time available for growth (incubation duration and nestling duration), and then, we examined the associations between nest DMR and growth rates. We focused on structural growth (tarsus length) for this analysis because mass and wing length can change after fledging (e.g., Martin et al., 2018), whereas skeletal growth is generally complete at fledging. We ran general linear models (GLMs) with nest DMR as the independent variable, and either incubation duration, nestling duration, posthatch growth rate, or postlay growth rate as the dependent variables. For estimates of nest DMR, we only used species for which we found at least 10 nests (16 species, Table 1) to avoid low sample size biases.

To determine whether species‐level growth rates (as described above) corresponded with *^r^p* among morphological traits at fledging, we ran general linear models and used an AICc model selection process to compare a suite of parameters linked to growth (Table 3). We included incubation and nestling durations (to assess whether estimates of “time to fledge” were important), posthatch and postlay growth rates for each morphological trait (i.e., wing‐adjusted, tarsus‐adjusted, and mass‐adjusted growth to determine whether somatic, skeletal, or wing growth were more important), the mean overall growth rates for the posthatch and postlay periods, mean trait size prior to fledge (to assess whether interspecific variation in size itself was more important than growth rates), and a null model. We ran each temporal, growth, and size parameter individually (as the independent variable) and included mean *^r^p* as the dependent variable. We also compared mean *^r^p* of cavity nesters to that of open‐cup nesters using a Student's *t*‐test to examine whether there were broad categorical differences by nest‐type. We used mean *^r^p* for species in which we had at least 10 individuals (16 species; Table 1) to avoid low sample size biases.

Additionally, we tested whether species‐level *^r^p* at fledging was linked to cumulative PFM rates using a linear regression with cumulative PFM rate as the independent variable and mean *^r^p* as the dependent variable. We ran the model with *^r^p* generated from *all* nestlings for the ten species we had postfledging data for, as well as from *just the individuals* we had postfledging data for; we excluded the Eastern Phoebe from the latter due to low sample sizes. To better understand the sources of postfledging mortality (i.e., predation versus exposure, disease), and whether this was important in influencing the relationship between *^r^p* at fledging and postfledging mortality, we partitioned postfledging mortality into “predator‐induced” and “non‐predator‐induced” sources, and compared the associations between species‐level mean trait covariance and both subsets of postfledging mortality, as well as cumulative postfledging mortality using an AICc model comparison approach.

For all models, we examined diagnostic plots to confirm that residuals approximated a normal distribution and met the assumption of homogeneous variances. In comparative methods, phylogenetic corrections are commonly used to control for perceived lack of statistical independence among species (Felsenstein, 1985; Pagel & Harvey, 1989). Though we pursued phylogenetically controlled analyses, we ultimately decided the uncorrected analyses were more appropriate for our questions given our framework and the limitations of our dataset (see Appendix 1 for details on our attempted analyses and rationale).

## 3 RESULTS

**FIGURE 2:**
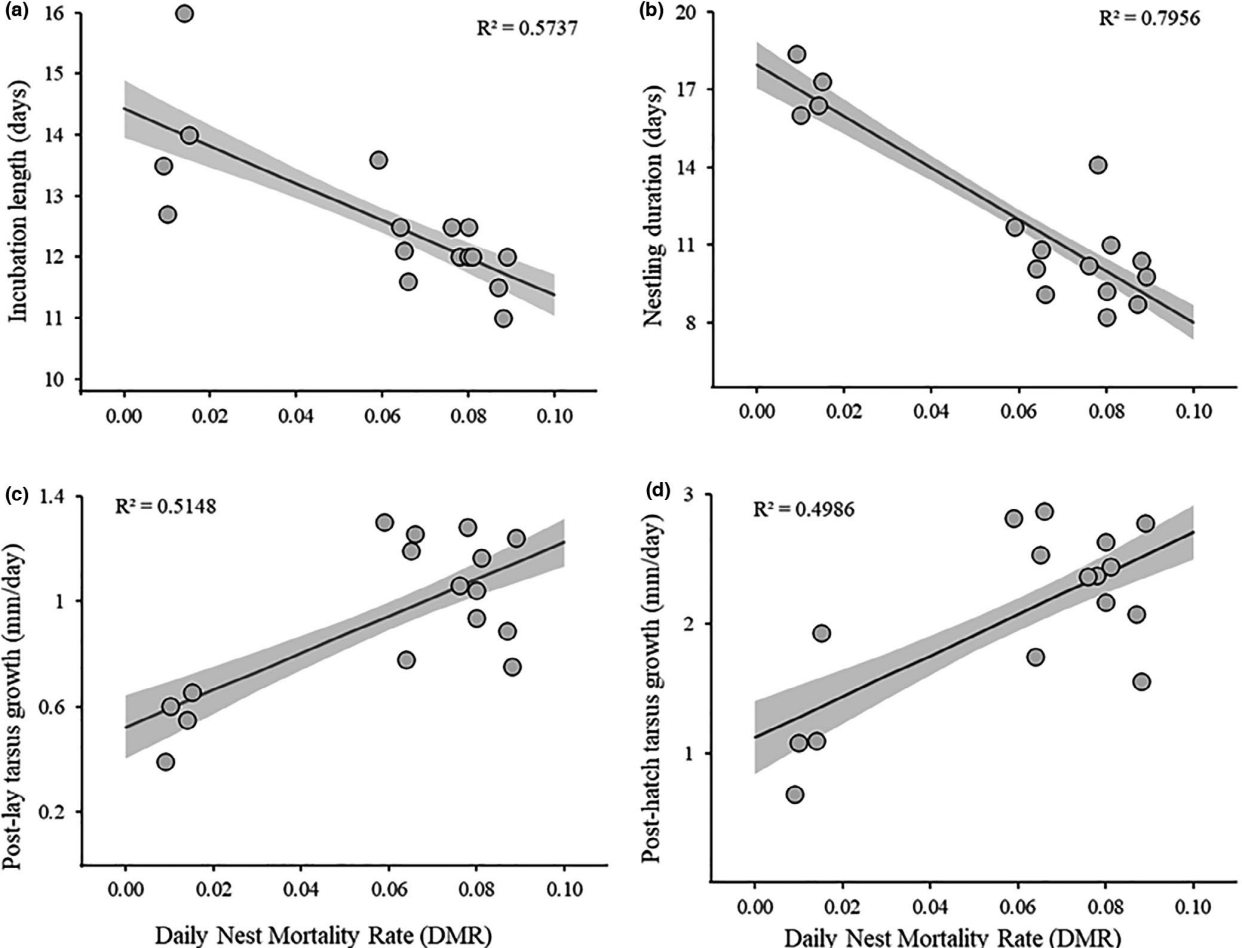
Graphical representation of associations between nest mortality risk and two measures of time to grow, and two measures of growth rate for 16 species of shrubland birds. Panels show the relationships between nest daily mortality rate and (a) egg incubation duration, (b) nestling duration, (c) tarsus growth postlay, and (d) tarsus growth posthatch. Egg incubation and nestling durations are given in mean number of days for each species. Tarsus growth rates were assessed by dividing the tarsus length at fledge by the number of days since the egg was laid (tarsus growth postlay) and number of days since the nestling hatched (tarsus growth posthatch). Shaded areas represent standard errors

**FIGURE 3:**
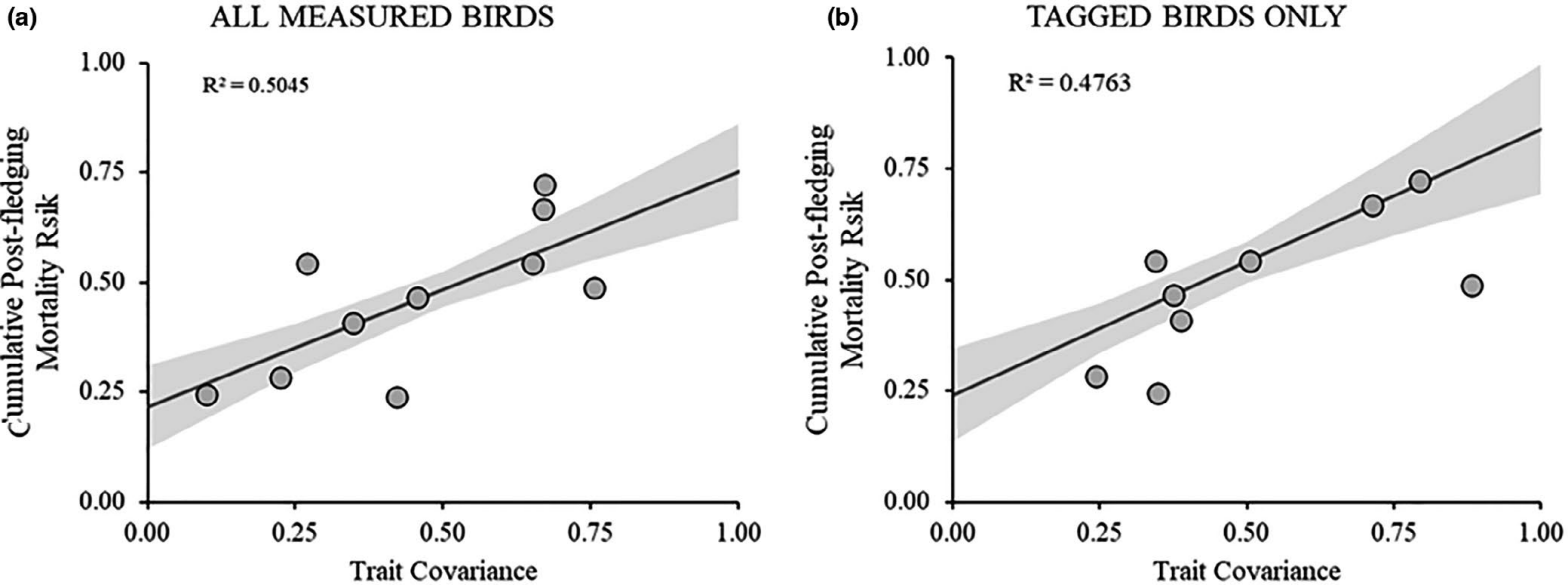
Associations between mean trait covariance level and postfledging mortality across nine shrubland bird species. Trait covariance is the species‐level mean correlation coefficient value for correlations among tarsus length, wing length, and mass at fledging. Panel a shows the relationship between mean trait covariance level and cumulative postfledging mortality rate using the correlation coefficient generated from all measured nestlings (1448 individuals), while panel b shows the relationship using the correlation coefficient generated from only the individuals outfitted with radio‐transmitters and used to calculate postfledging mortality (369 individuals). Shaded areas represent standard errors

Daily nest mortality was significantly inversely associated with both incubation duration and nestling duration across species (Table 2; Figure 2a, b), although the association was substantially stronger for nestling duration (Table 2). Similarly, nest mortality was significantly positively associated with postlay tarsus growth as well as posthatch tarsus growth (Table 2; Figure 2c, d), although the association was stronger for posthatch growth. For associations between growth rates and trait covariance strength, we found that multiple components of growth and duration of time to grow were positively associated with trait covariance (Table 3). Cavity nesters, which generally experience reduced nest mortality and fledge at substantially older ages than open‐cup nesting species, had significantly lower levels of trait covariance than open‐cup nesters (cavity nester trait covariance = 0.199, open‐cup nester trait covariance = 0.467; *t* = −2.23, *p* = .042). We also found positive associations between trait covariance and postfledging mortality—for the nine species in which we tagged nestlings—for trait covariance values generated from all nestlings measured (1448 individuals; *n* = 10; *F* = 8.14, *β* = 0.535 ± 0.19 [SE], *p* = .021; Figure 3a), as well as trait covariance values generated from only the individuals that were tagged and followed postfledge (369 individuals; *n* = 9; *F* = 6.37, *β* = 0.797 ± 0.32 [SE], *p* = .040; Figure 3b) (Table 1). When we partitioned postfledging mortality into “predator‐induced” and “non‐predator‐induced” sources, we found that neither subset was significantly associated with mean trait covariance in contrast to the strong association between mean trait covariance and cumulative postfledging mortality (Table 4). Neither source of mortality alone outperformed the null, although non‐predator‐induced mortality performed significantly better than predator‐induced mortality (Table 4).

**TABLE 2:** Association between nest mortality rate and growth periods for 16 shrubland bird species

Dependent variable	Estimate	SE	*N*	*F*	*p*
Incubation duration	−30.46	7.02	17	18.84	<.001
Nestling duration	−99.27	13.45	17	54.49	<.001
Posthatch tarsus growth	15.84	4.25	17	13.92	.002
Postlay tarsus growth	7.01	1.82	17	14.85	.002

Note

Results of linear regressions in which nest daily mortality rate was the independent variable and measures of time or growth were the dependent variables. Incubation duration is the mean number of days from lay to hatch, and nestling duration is the mean number of days from hatch to fledge. Posthatch tarsus growth represents a size‐corrected rate of growth over the nestling period and is calculated by dividing the tarsus length on the day of fledging by the nestling duration. Postlay tarsus growth represents a size‐corrected rate of growth over the entire nest period and includes embryonic growth as well as nestling growth. This value is calculated by dividing the tarsus length at fledge by the total number of days in the nest (incubation and nestling periods).

**TABLE 3:** AICc model comparisons for associations between measures of growth and trait covariance strength across 16 shrubland bird species

Model	AICc	ΔAICc	w*_i_*	Estimate	*p*‐value
**Nestling period duration**	**−56.27**	**0.00**	**0.24**	**−0.035**	.**013**
**Total nest duration**	**−55.72**	**0.56**	**0.18**	**−0.027**	.**018**
**Postlay tarsus growth**	**−55.50**	**0.77**	**0.17**	**0.402**	.**019**
**Posthatch wing growth**	**−54.58**	**1.69**	**0.10**	**0.113**	.**031**
**Posthatch tarsus growth**	**−53.86**	**2.41**	**0.07**	**0.146**	.**044**
Posthatch mass growth	−52.71	3.56	0.04	0.098	.079
Mean tarsus length	−52.68	3.60	0.04	0.017	.081
Posthatch total growth	−52.65	3.62	0.04	0.113	.082
Postlay mass growth	−52.06	4.21	0.03	0.198	.112
Null	−51.69	4.58	0.02	0.419	—
Postlay wing growth	−51.21	5.06	0.02	0.177	.180
Mean mass	−50.21	6.07	0.01	0.005	.332
Postlay total growth	−50.07	6.20	0.01	0.148	.363
Mean wing length	−49.51	6.76	0.01	−0.003	.555

Note

Results from linear regressions examining associations between measures of growth and time to grow, and mean trait covariance strength for associations among mass, tarsus length, and wing length. Mean trait covariance was the dependent variable in each model, and morphometric traits, temporal parameters, or size‐adjusted growth rates were the independent variables. Morphometric traits (e.g., mean mass) were assessed on the day of fledging, time to grow (nestling duration and total period postlay) reflects the temporal constraints on growth, and posthatch or postlay measures reflect growth that occurred during the nestling period (posthatch) or during the embryonic and nestling periods (postlay). Total size measures are the average values across mass, wing length, and tarsus length. All models are presented relative to the null model, and those rows in bold are those in which the parameter performed better than 2 ΔAICc compared to the null.

**TABLE 4:** AICc model comparison among sources of postfledging mortality and trait covariance strength across nine shrubland bird species

Model	AICc	Delta AICc	Model weight
Cumulative Postfledging Mortality	−32.36	0.00	0.64
Nonpredation Postfledging Mortality	−30.24	2.12	0.22
Null	−28.55	3.81	0.10
Predation Postfledging Mortality	−26.92	5.43	0.04

Note

Linear regression models comparing sources of postfledging mortality for the nine species of shrubland birds in which postfledging mortality was assessed using radio‐transmitters over the first 28 days following fledging. Trait covariance strength among mass, wing length, and tarsus length was the dependent variable, and sources of mortality were the independent variables. Mortality was partitioned into predation, non‐predation‐based, and the sum of the two (cumulative), and these were compared against a null model. Cumulative mortality was the only model to significantly outperform the null.

## 4 DISCUSSION

**FIGURE 4:**
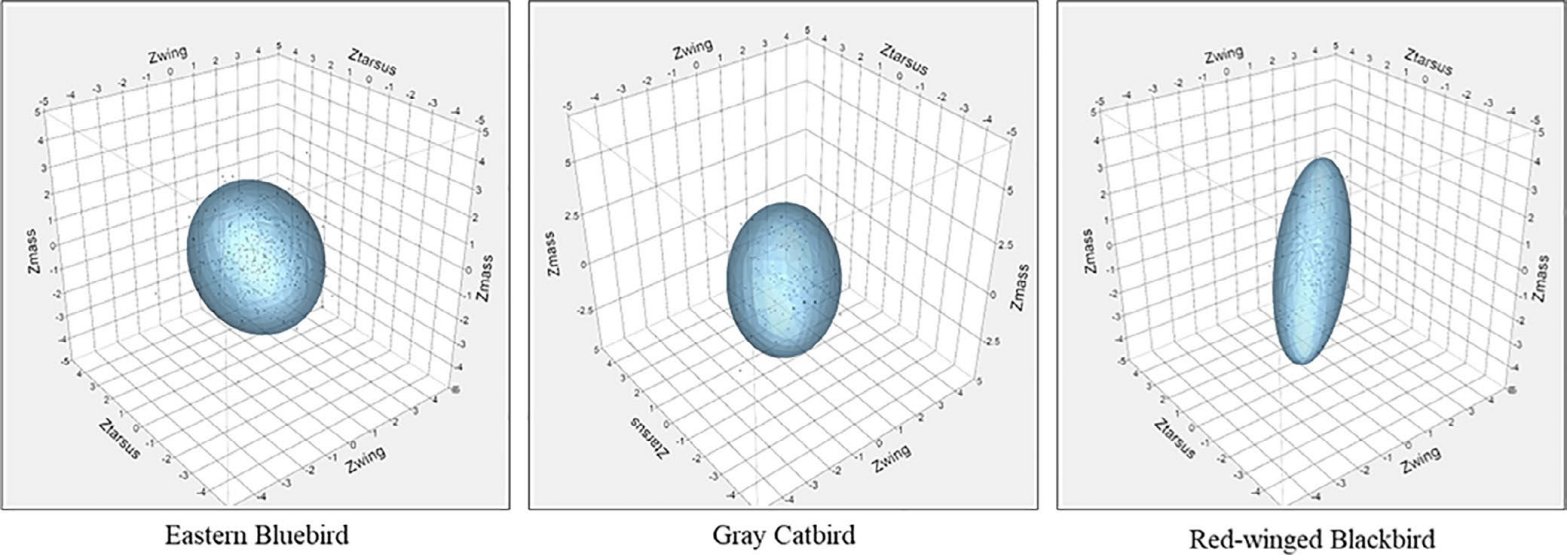
Three‐dimensional depiction of multivariate trait space for three shrubland bird species that exhibit low, medium, and high levels of trait covariance strength. Morphometric data for tarsus length, wing length, and mass were standardized (Std) for comparisons across species using Z transformations. The three species included here are representative of species with low mean levels of trait covariance (eastern bluebird; mean *r* = 0.10; *n* = 348), moderate levels (gray catbird; mean *r* = 0.27; *n* = 95), and high levels (red‐winged blackbird; mean *r* = 0.76; *n* = 104). Normal contour ellipsoids contain 90% of the data points for each species

We found evidence indicating that nest predation risk is strongly tied to interspecific rates of growth in a community of passerine birds, which aligns with previous research (Bosque & Bosque, 1995; Martin et al., 2018; Remeš, 2007; Remeš et al., 2020; Ton & Martin, 2020) and supports our predictions (Figure 1). We also found that rates of growth correlated with levels of morphological trait covariance in which species with more rapid growth exhibited stronger trait covariance values at the time of fledging. These trait covariance values predicted rates of mortality in the next life stage, wherein those species with stronger trait covariance had higher rates of postfledging mortality than species with weaker trait covariance. Together, these findings provide an indication that there are costs associated with rapid growth among species, and that these costs may be expressed as an increase in phenotypic canalization (i.e., higher trait covariance strength) (Figure 4), and increased mortality in the next life stage.

For this study, we sought to examine whether there were costs associated with more rapid growth that would be apparent among species, and whether those costs would manifest as stronger trait covariance. Our results suggest that the same physiological processes responsible for tighter trait covariance at the intraspecific level may be operating at the interspecific level, and that rapid growth may incur universal costs (i.e., across taxa). Species that exhibit more rapid rates of growth presumably have evolved mechanisms to mitigate the physiological costs of fast growth (sensu Metcalfe & Monaghan, 2003), but there are likely limits on a species’ ability to cope with these costs. Indeed, organisms that grow relatively faster (at both the within‐ and among‐species levels) generally have shorter life spans than those that grow and develop more slowly (intraspecific (Janssens & Stoks, 2018; Lee et al., 2013; Olsson & Shine, 2002), interspecific (Ricklefs, 2006; Rollo, 2002)). Furthermore, the patterns we documented between nest daily mortality rates and incubation and nestling periods indicate that species under relaxed nest predation risk may have evolved longer incubation and nestling periods to allow for slower rates of growth, higher quality phenotypes at fledging (e.g., slower growth often leads to higher quality feathers; Callan et al., 2019), and higher survival upon leaving the nest (Jones & Ward, 2020; Martin et al., 2018).

Alternatively (but not mutually exclusively), species under higher nest predation risk may have been forced to reduce their incubation and nestling periods and accelerate growth (Bosque & Bosque, 1995; Martin, 1995; Remeŝ & Martin, 2002; Remeš et al., 2020). Of the four temporal and growth measures we examined, nestling duration was by far the most strongly associated with nest predation risk. In general, we found that the posthatch measures (i.e., nestling duration, posthatch growth) were more strongly linked to nest predation and trait covariance strength than the postlay measures. These results suggest that predation risk more strongly impacted the nestling period than the incubation period, and subsequently that posthatch growth was more important for shaping levels of trait covariance and postfledging mortality than postlay growth. These findings are consistent with studies providing compelling links among nest mortality, nestling period length, and subsequent postfledging survival (Jones & Ward, 2020; Martin et al., 2018; Remeš & Matysioková, 2016). To be clear, we are not suggesting that growth and development that occur in the egg are unimportant, but rather that the variation in nest predation, growth, and condition in our study was driven more strongly by extrinsic and intrinsic factors acting during the nestling period. Indeed, this follows previous work, which has shown that bird embryos are of similar size across species and are expected to experience similar growth rates during the early phases of development and growth, and that interspecific variation in growth mostly occurs during the later stages of development (Cooney et al., 2020; Von Bertalanffy, 1957).

Growth is the increase of mass over time for a given tissue and depends upon cell size increases and proliferation, whereas development is the differentiation of soma. The two processes exhibit considerable temporal overlap during the early‐life period for many organisms (Cooney et al., 2020). Unfortunately, we could not sufficiently detangle the two processes in this study, although there is circumstantial evidence that growth may be more important than development for shaping *^r^p*. For example, the embryo (egg stage) and nestling both undergo growth and development, but the posthatch level of growth far exceeds that which occurs in the egg (Cooney et al., 2020). Conversely, the embryonic period is when a large proportion of development occurs. In our analyses examining which aspects of growth and temporal periods were more strongly associated with *^r^p*, we found that with the exception of tarsus growth, all “posthatch only” models outperformed “postlay only models” and that all “posthatch only” models outperformed the null, in contrast to just two “postlay only” models (Table 3). These results suggest that the posthatch period alone was more important in shaping trait covariance than the postlay period (egg incubation and posthatch periods together) and indicate that posthatch growth may play a larger role than development in determining trait covariance strength. If most interspecific variation in growth occurs during the latter stages of development (sensu Cooney et al., 2020; Von Bertalanffy, 1957), it follows that species‐level differences in the effects of growth‐related stress would be linked to this phase of growth and development.

Another important component to these analyses is that our measures of time and growth provide very coarse, conservative estimates of growth rates for each species. Due to the logistical constraints, nestlings were only measured once, and thus, true measures of posthatch growth (i.e., longitudinal data; Ricklefs, 2010) were unavailable. However, despite the fact that larger eggs generally hatch larger chicks (Perrins, 1996) and that the proportional amount of growth from posthatch to fledge may differ, none of the body size traits alone were significant in predicting interspecific variation in *^r^p*, and the posthatch period of growth emerged as the driving force for variation in *^r^p* (Table 3). As with past research, we also found that nest DMR was strongly inversely associated with the nestling duration (Bosque & Bosque, 1995; Martin, 2015; Martin et al., 2018), and positively associated with posthatch tarsus growth (Figure 2). Together these results, and work by others (see Martin, 2015; Martin et al., 2018; Ricklefs et al., 2017) indicate that our coarse estimates of growth were biologically meaningful and suggest that more precise estimates of growth (Ricklefs, 2010) may provide even stronger associations.

Previous work on postfledging mortality indicates that wing development/growth may be associated with postfledging mortality at both the intraspecific and interspecific levels (Jones & Ward, 2020; Jones et al., 2017; Mainwaring, 2016; Martin et al., 2018). The putative source of this mortality is predation, and the relationship between wing development and predation is based on the theory that fledglings with more developed wings should be better able to escape predators (Jones et al., 2020; Jones & Ward, 2020; Martin et al., 2018; Remeš & Matysioková, 2016). This is undoubtedly true for many species, but our results suggest that there are other factors which mediate postfledging survival. Postfledging mortality should be the product of various factors, such as exposure (e.g., extreme temperatures and weather events), starvation, and disease, in addition to predation (Jones et al., 2017). Furthermore, the probability of being depredated can increase for individuals that are sick or otherwise in poor body condition (Hudson et al., 1992, Wirsing et al., 2002). Our analysis of predator‐induced mortality versus non‐predator‐induced mortality found that neither subset was significantly associated with mean trait covariance in contrast to the strong association between mean trait covariance and cumulative postfledging mortality (Table 4). Neither source of mortality alone outperformed the null, although non‐predator‐induced mortality performed significantly better than predator‐induced mortality (Table 4). These results indicate that even though predator‐induced mortality is the largest source of mortality for fledglings (summarized in Cox et al., 2014), a fledgling's probability of being depredated may be influenced by other factors related to their somatic or epigenetic state that are reflected in mean trait covariance values. In addition, recent work documenting positive associations between trait covariance strength and mortality in a laboratory population of zebra finches with no predation (Merrill & Grindstaff, 2018) and among American robin (*Turdus migratorius*) nestlings prior to fledging (Ospina et al. *unpublished data*) suggests that trait associations are themselves indicative of processes that impact an organism's survival.

Another important consequence of tighter trait covariance is that it is indicative of a reduction in the volume of multivariate trait space (Figure 4), presumably reflecting more canalized development and reduced phenotypic flexibility (Gianoli & Palacio‐Lopez, 2009; Merrill & Grindstaff, 2018; Van Dongen, 2006). It remains unclear, however, whether these changes in trait covariance are adaptive, “making the best of a bad situation,” or simply costs associated with challenging early‐life conditions. These scenarios do not have to be mutually exclusive, but the elevated mortality linked to higher trait covariance presented here and in previous work (Merrill & Grindstaff, 2018, Ospina et al. *unpublished data*) suggests a high cost. If rapid growth leads to more canalized development, organisms that grow rapidly may have reduced capacity for dealing with future challenges due to lost phenotypic flexibility, which may manifest in lower survival during subsequent juvenile and adult life stages (Jones & Ward, 2020; Remeš, 2007). This loss of flexibility may therefore pose a major constraint on rates of growth at both the intra‐ and interspecific levels.

## CONFLICT OF INTEREST

Authors declare no competing interests.

## AUTHOR CONTRIBUTION

**Loren Merrill:** Conceptualization (lead); Data curation (supporting); Formal analysis (lead); Writing‐original draft (lead); Writing‐review & editing (equal). **Todd M. Jones:** Conceptualization (supporting); Data curation (lead); Formal analysis (supporting); Funding acquisition (lead); Writing‐original draft (supporting); Writing‐review & editing (equal). **Jeffrey D. Brawn:** Conceptualization (supporting); Data curation (supporting); Funding acquisition (supporting); Writing‐original draft (supporting); Writing‐review & editing (equal). **Michael P. Ward:** Conceptualization (supporting); Data curation (supporting); Funding acquisition (lead); Writing‐original draft (supporting); Writing‐review & editing (equal).

## Figures and Tables

**FIGURE A1:**
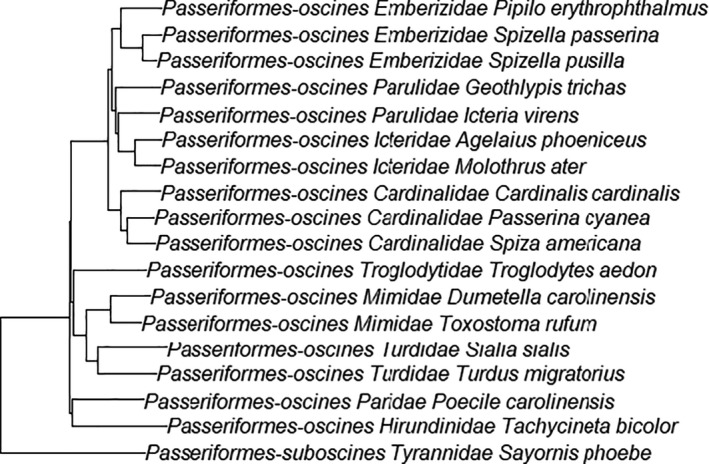
Phylogenetic relationships of species used in our statistical models. Under phylogenetic generalized least squares analyses, the species dataset did not produce enough information to provide accurate estimates of phylogenetic signal (λ)

## Data Availability

Data for this article are available at the Illinois Databank: https://doi.org/10.13012/B2IDB‐8719858_V1

## APPENDIX 1

Methods: Many studies have demonstrated that phylogenetic relatedness is not a widespread bias among ecological research and argue that phylogenetic corrections should only be applied where it is conceptually appropriate (de Bello et al., 2015; Losos, 2011). Given that our study examines community trait composition in relation to environmental filtering by mortality (de Bello et al., 2015), is among a small group of closely related species (Boettiger et al., 2012), and that similar traits have exhibited no phylogenetic signal (e.g. nestling period length; Losos, 2011), we feel applying phylogenetic corrections is inappropriate in this case. Furthermore, we would argue that models that control for phylogeny do not focus on the questions we are trying to answer (i.e., differences in results from phylogenetically controlled models would not invalidate our findings, rather answer a different question; sensu de Bello et al., 2015). Regardless, no specific guidelines currently exist for when to apply phylogenetic corrections (de Bello et al., 2015). Thus, we attempted to correct for phylogenetic effects using phylogenetic generalized least squares (PGLS) analyses. Following Burleigh et al. (2015), we derived a consensus phylogenetic tree (Figure A1) and conducted a PGLS analysis with the Caper package (Orme, 2018) in R v3.5.2 (R Development Core Team) to test for a phylogenetic signal (λ). Not surprisingly, our dataset of 21 species did not provide enough information to derive a reliable estimate of λ, as lower numbers of taxa often lack statistical power and are unable to produce accurate estimates of phylogenetic signal (Boettiger et al., 2012). Similar to Brawn et al. (2017), our results suggest that there is likely little to no phylogenetic signal in our dataset. Thus, given that (1) applying phylogenetic corrections may be conceptually inappropriate in this case (sensu de Bello et al., 2015; Losos, 2011) (2) that phylogenetic signal is likely low in our dataset (Brawn et al., 2017); and (3) that applying phylogenetic corrections without adequate assessment of phylogenetic signal can be inappropriate or misleading (Revell, 2012), we deferred to results from our original analyses.
